# Performance of ceramic passive samplers for the monitoring of PFAS in surface water and groundwaters

**DOI:** 10.1007/s00216-026-06533-y

**Published:** 2026-05-08

**Authors:** Giacomo Moro, Sergio Santana-Viera, Sandra Pérez, Silvia Lacorte

**Affiliations:** 1 Department of Environmental Chemistry, Institute of Environmental Assessment and Water Research (IDAEA-CSIC), Barcelona, Spain; 2 Doctoral Programme in Analytical Chemistry and the Environment, University of Barcelona, Barcelona, Spain

**Keywords:** PFAS, Passive sampling, Water, Sampling rate, Blank analysis

## Abstract

Per- and polyfluoroalkyl substances (PFAS) are persistent environmental contaminants widely detected in aquatic systems, yet their accurate quantification remains challenging due to strong temporal variability and ultra-trace concentration levels. Ceramic passive samplers (CPSs) represent a novel and promising alternative to conventional sampling techniques, offering time-integrated measurements while minimizing the influence of short-term concentration fluctuations. In this study, CPS devices were used in combination with liquid-chromatography/tandem mass spectrometry to determine PFAS in groundwater and river water within the Besòs River catchment area (Barcelona, Spain). First, CPS were calibrated for seven target PFAS under controlled laboratory conditions. All compounds exhibited linear uptake behavior throughout a 20-day calibration period, allowing the determination of compound-specific sampling rates. Laboratory blank experiments were also performed to evaluate background contamination and ensure reliable quantitative interpretation of field data. The calibrated CPSs were subsequently deployed in both groundwater and river water for 14 days. Total PFAS concentrations ranged from 19.7 to 250 ng L^−1^, revealing widespread contamination across the study area. The concentrations obtained were consistent with those reported previously for the Besòs system and for other impacted European aquatic environments using conventional grab sampling methods. These results demonstrate the strong potential of CPS as an innovative and robust tool for quantitative PFAS monitoring in surface water and groundwater, providing reliable time-integrated concentration data and supporting their application in long-term environmental surveillance programs.

**Graphical Abstract:**

**Figure:**
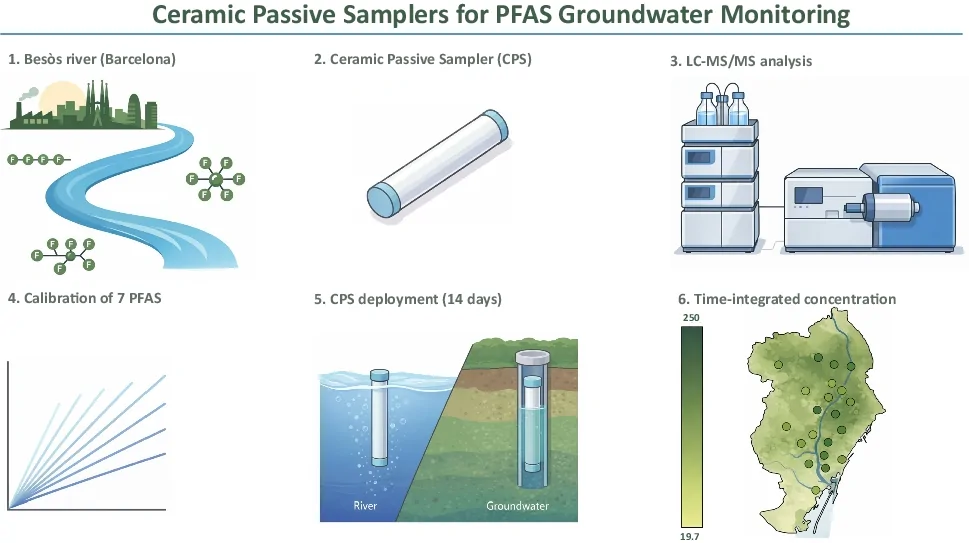

**Supplementary Information:**

The online version contains supplementary material available at https://doi.org/10.1007/s00216-026-06533-y.

## Introduction

Per- and polyfluoroalkyl substances (PFAS) are a large class of synthetic organofluorine compounds widely used for their chemical stability, surfactant properties, and resistance to thermal and biological degradation [1–3]. These characteristics result in extreme environmental persistence and limited natural attenuation, leading to their widespread occurrence in aquatic systems worldwide. Military and firefighting training facilities, airports, PFAS manufacturing plants and wastewater treatment plants are main sources of PFAS to receiving waters [4]. Groundwater (GW) is of particular concern, as many PFAS exhibit high mobility under subsurface conditions and may contaminate groundwater, which represents a major source of drinking water in many regions [5].

Regulatory efforts have historically focused on legacy long-chain PFAS, notably perfluorooctanoic acid (PFOA) and perfluorooctane sulfonate (PFOS), which were phased out in many jurisdictions during the 2000 s due to their persistence, bioaccumulation and toxicity [6, 7]. Despite these measures, PFOA and PFOS remain frequently detected in the soil-groundwater system as a consequence of historical emissions and long residence times [5, 8]. At the same time, the transition away from long-chain PFAS has led to increased use of shorter-chain alternatives, including perfluorohexanoic acid (PFHxA), perfluorohexanesulfonate (PFHxS), and perfluorobutanesulfonate (PFBS), which are still expected to pose an environmental risk despite the lack of conclusive evidence [9]. This calls for the development of new techniques to measure their concentration in groundwater and river water in a representative and accurate way. Measuring them through grab sampling poses limitations, as it is a punctual sampling and therefore susceptible to spikes in concentration [10]. In this context, passive sampling techniques represent a promising complementary approach to conventional grab sampling for the assessment of PFAS occurrence in groundwater [11].

The most established passive sampler used to measure PFAS in water is polar organic chemical integrative sampler (POCIS), with a variety of sorbents such as hydrophilic-lipophilic balance (Oasis HLB) [12–14], weak-anion exchange (Oasis WAX) [12, 15], silica-immobilized imidazolium-based ionic liquids (IIL) [13], and mixed-mode weak-anion exchange (Strata X-AW) [16–18]. Alternative samplers include microporous polyethylene tubes (PE) filled with Strata X-AW [19] and diffusive gradients in thin hydrogel films (DGT) [20]. Recently, a type of ceramic dosimeter named ceramic filter tubes (CFT) filled with a polyacrylamide gel and WAX sorbent was used to measure PFAS in wastewater [21].

Ceramic passive samplers (CPSs) are a relatively novel type of ceramic dosimeter optimized to achieve high porosity and surface area [22]. They have been used before to measure anticancer drugs in wastewater influent and effluent water [23], pharmaceuticals and drugs of abuse in river and drinking water [24], and chlorophenoxy pesticides in landfill-contaminated groundwater [25].

In this work, CPSs’ deployment was optimized to measure low PFAS concentrations in ground- and river water. The study site is the lower course of the Besòs River, close to the densely urbanized Barcelona city (Spain). This basin historically was very industrialized and during the 1990 s, many old industrial plants were cleared and the area was urbanized, but chemical industries, plastic, leather, textile, paper, food and metallurgical industries still remain, and represent a source of PFAS to GW. In a first step, a calibration experiment was carried out to determine the diffusion coefficient (De) and sampling rate (Rs) of 7 common PFAS. Their Rs were compared with established passive samplers. Then, CPSs were deployed in 14 groundwater wells and 2 river sites in the lower course of the Besòs River. The calculated Rs values were used to estimate PFAS concentrations in the sampled waters. A separate quality-control study was carried out to investigate potential PFAS contributions from consumables, solvents, and other sources of laboratory background. The potential of CPS for PFAS monitoring in GW is discussed.

## Experimental

### Materials and chemicals

Seven common PFAS, namely perfluorobutanesulfonate (PFBS), perfluorohexanoic acid (PFHxA), perfluorohexanesulfonate (PFHxS), linear perfluorooctanesulfonate (n-PFOS), perfluorooctanoic acid (PFOA), perfluorononanoic acid (PFNA) and perfluorododecanoic acid (PFDoA) (> 98%) were purchased from Sigma-Aldrich (Steinheim, Germany). Individual stock solutions of the seven target PFAS were prepared in methanol at a concentration of 100 mg L^−1^ and stored at − 20 °C. These standards were used for CPS calibration. Two isotopically labelled PFAS compounds used as internal standards (^13^C_4_-PFOS and ^13^C_4_-PFOA) were obtained from Wellington Laboratories (Ontario, Canada). A working solution of the internal standards (1 mg L^−1^ in methanol) was prepared for addition to samples and calibration standards. Calibration standards for the 7 PFAS were prepared from a PFAC-MXH standard mixture (Wellington Laboratories, Ontario, Canada) at concentrations ranging from 1 to 4 µg L^−1^.

For chromatographic analysis, high-purity acetonitrile, methanol and water (HPLC grade) were purchased from Merck (Darmstadt, Germany). Ammonium acetate (> 96%, ACS grade) was obtained from Sigma-Aldrich. For CPS cleaning, conditioning and extraction, methanol (HPLC grade) and ethanol (> 95%, reagent grade) obtained from Merck were used. Ultrapure water was produced on site using a Milli-Q water purification system.

Ceramic passive samplers (CPSs) were produced in-house by slip casting followed by sintering at 1300 °C, as previously described [23]. The CPSs used are highly porous ceramic cylinders with 42 mm length, 16 mm external diameter, 12.6 mm internal diameter and 1.7 mm wall thickness. They were closed with natural thermoplastic elastomer side-pull caps, in sizes 15.5–13.5 mm (part number: 10275986-494305) purchased from Essentra (Kidlington, Oxfordshire, UK).

### CPS preparation and calibration

CPSs were prepared by filling each sampler with 300 mg of Oasis HLB and 100 mg of Porapak RXN RP as sorbent material (both from Waters Corp., Milford, MA, USA). The combination of both sorbents enables the retention of more and less polar contaminants [24]. The sorbent mixture was conditioned using a 50:50 (v/v) water–methanol solution, which was also used to fill the CPS prior to capping. The CPSs were then left in a glass jar with Milli-Q water until deployment (generally the next day).

Calibration for seven PFAS was carried out, to determine the uptake rates (k), the effective diffusion coefficients (De, Eq. 1) and the sampling rates (Rs, Eq. 2).

1 $$D_e=\frac{k\cdot L}{C_w\cdot A}$$

2 $${\mathrm{R}}_{\mathrm{s}}=\frac{\mathrm{k}}{{\mathrm{C}}_{\mathrm{w}}}$$

where *k* (ng day^−1^) is the uptake rate, *L* is the CPS wall thickness (1.7 mm), *C*_*w*_ is the water concentration and* A* is the CPS surface area (18.85 cm^2^).

For CPS calibration, 20 CPSs were tied in pairs with fishing line and immersed in a glass beaker with 4 L of mineral water spiked at a concentration of 500 ng L^−1 ^with the seven target PFAS. Mild agitation was applied with an Orbital shaker at 50 rpm to simulate groundwater conditions. In parallel, eight CPSs were placed in 1 L of mineral water without spiking, to serve as calibration blanks. Both vessels were covered with aluminium foil to prevent photodegradation. Calibration was performed at room temperature (21-23^o^C).

The calibration experiment lasted 20 days, based on preliminary data implying that accumulation would be linear over a period of 1 month [24]. CPSs were retrieved in duplicate at days 0, 1, 2, 5, 7, 9, 12, 14, 16, 19 and 20 for the spiked experiment and 0, 7, 14 and 20 for the blank experiment, followed by extraction as described in “Sample extraction”.

### Blank contribution

To assess potential sources of PFAS background related to laboratory procedures, a dedicated screening experiment was conducted. Materials potentially in contact with samples or solvents were extracted, including bulk sorbents (300 mg Oasis HLB and 100 mg Porapak), CPS caps, glass vials and caps, aluminium foil, nitrile gloves, pipette tips, and the septum of a methanol bottle. All materials were extracted using 10 mL of methanol and subsequently concentrated under a gentle stream of nitrogen following the same procedure applied to CPS sorbents (“Sample extraction”).

Procedural blanks were prepared in parallel using methanol taken directly from the original bottle as well as methanol dispensed through laboratory solvent dispensers. Internal standards and buffer solutions identical to those used for CPS samples were added to all blanks and material extracts at a concentration of 25 µg L^−1^ and 10 mM respectively.

To assess background contributions associated with field handling and transport, four additional CPSs were prepared. These CPSs were transported to the field sites in glass containers together with the deployed CPSs and handled identically, but were not deployed in the field.

### Field deployment in the Besòs River basin

**Fig. 1:**
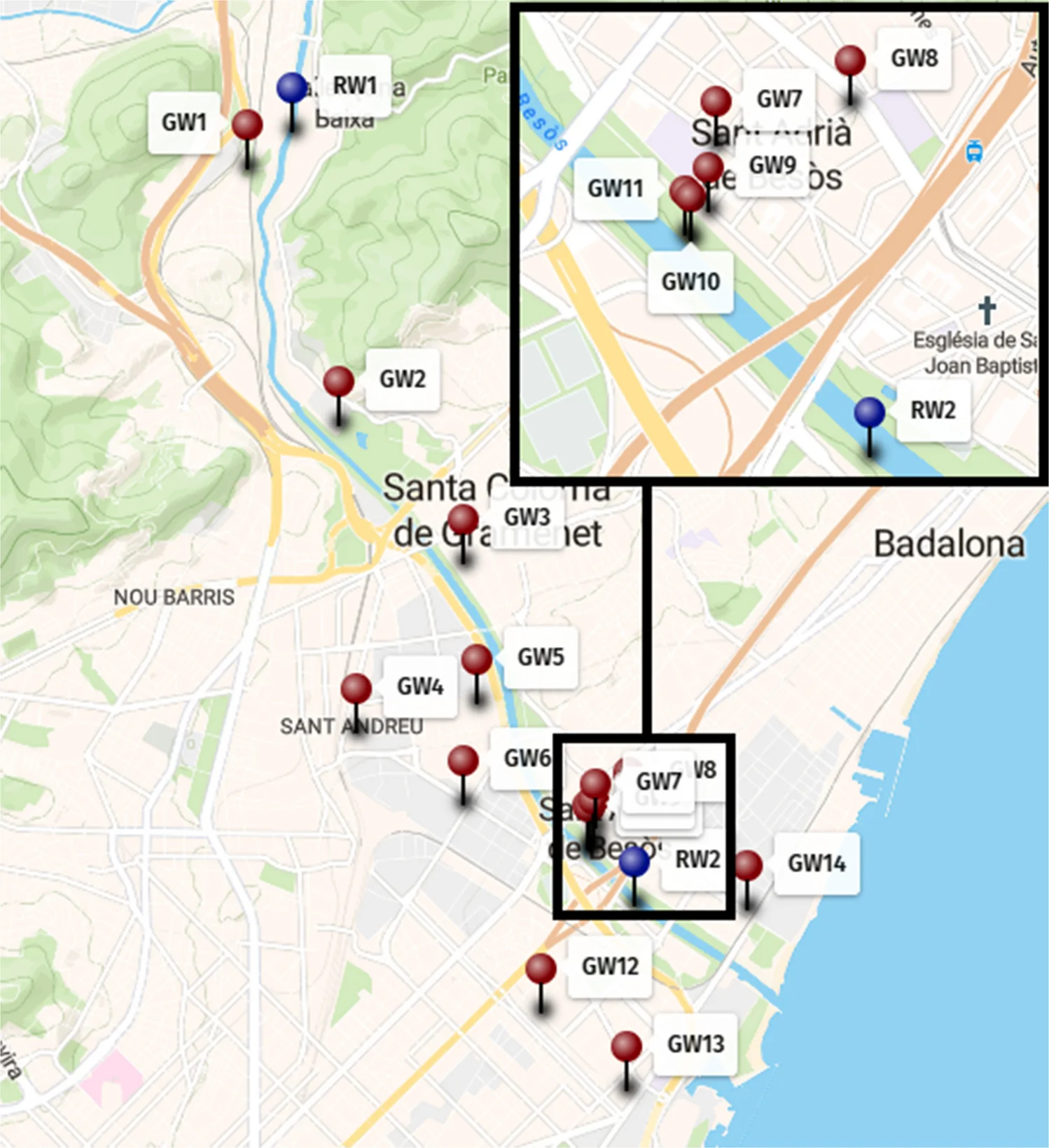
CPS deployment sites in the Besòs River basin (Barcelona, Spain). GW1–GW14: well sampling locations. RW1 and RW2: river locations. Codes are from upriver to the river mouth and comprise the last 13 km of the Besòs River. Base map: © OpenStreetMap contributors, ODbL ( https://www.openstreetmap.org/copyright)

Sampling was performed in 14 groundwater wells (piezometers) and two river sites along the Besòs River in the Metropolitan Area of Barcelona, Spain, during January 2025. The Besòs River basin is a known contamination hotspot due to the high population density and presence of several wastewater treatment plants, hospitals and industrial activities [26]. Sampling locations are reported in Fig. 1. CPSs were enclosed in a polyester mesh and deployed in piezometers or river water for a period of 14 days. Following retrieval, CPSs were wrapped in aluminium foil, stored refrigerated, and extracted on the same or following day. The concentration in water was calculated according to Eq. 3:

3 $$\mathrm{Cw}=\frac{\mathrm{M}}{\mathrm{Rs}\cdot \mathrm{t}}$$

where M is the mass of each PFAS accumulated in the CPS during the deployment period, Rs is the calculated sampling rate and t is the deployment time (14 days).

### Sample extraction

CPS extraction from the calibration experiment and field deployments was done using the same procedure. Sorbent material was transferred to 15-mL polypropylene centrifuge tubes and CPS were rinsed with approximately 10 mL of Milli-Q water to recover all sorbent from the CPS. After centrifugation at 4000 rpm for 10 min, the aqueous supernatant was discarded. Methanol (10 mL) was added to the sorbent, followed by vortexing and sonication for 10 min. This step was repeated three times without solvent replacement. After centrifugation (same conditions), the methanolic extract was transferred to a clean 40 mL glass tube and evaporated to dryness at 40 °C under a gentle nitrogen stream. The residue was reconstituted in 200 µL methanol and 200 µL water, vortexed, and centrifuged to remove suspended solids. An aliquot of 100 µL was transferred to a chromatographic vial, and 10 ng internal standard solution (10 µL of 1 mg L^−1^ solution) and 10 µL ammonium acetate buffer (125 mM) were added, yielding a final buffer concentration of 10 mM.

### Liquid chromatography–mass spectrometry analysis

Analysis was performed using an Acquity ultra-high-performance liquid chromatography coupled to a XEVO TQ-S triple-quadrupole mass spectrometer (Waters, USA) through a heated electrospray ionization source operated in the negative mode (HESI-). Chromatographic separation was achieved using a Waters Acquity UPLC BEH C18 column (2.1 × 100 mm, 1.7 µm particle size). A Waters XBridge Phenyl delay column (4.6 × 50 mm, 3.5 µm particle size) was installed between the solvent mixer and the autosampler, to retain and delay background PFAS originating from the instrument and mobile phase.

The mobile phase consisted of (A) water and (B) methanol–acetonitrile (80:20, v/v), both containing 10 mM ammonium acetate as buffer. The gradient started at 50% B for 3 min, increased linearly to 100% B over 7 min, was held for 4 min, returned to 50% B over 2 min, and equilibrated for 2 min. The flow rate was 0.3 mL min^−1^, column temperature 30 °C, and injection volume 10 µL.

The heated electrospray source was operated with a capillary voltage of 2.1 kV (negative), temperature of 150 °C and desolvation temperature of 500 °C. Nitrogen was used as cone and desolvation gas at flow rates of 150 and 1000 L h^−1^, respectively. Argon was used as collision gas at 0.19 mL min^−1^. Data acquisition was performed using the MassLynx 4.1 software package, and version 4.2 with the QuanLynx add-on was used for data processing.

A selected reaction monitoring (SRM) method (Table 1) was used to measure the seven PFAS of interest. Calibration curves were constructed over the range 0.1–200 µg L^−1^ (eight points) in 1 mL of 50:50 water–methanol containing 10 mM ammonium acetate and 25 ng internal standards (25 µL of 1 mg L^−1^ solution). Weighted (1/x) linear regression was used. Internal standard quantification was applied using 13C_4_-PFOS for perfluoroalkanesulfonates and 13C_4_-PFOA for perfluoroalkylcarboxylic acids. Method Quantification Limits (MQLs) were calculated using the lowest instrumental calibration point (0.1 µg L^−1^) multiplied by the volume of the extract (400 µL) and divided by the Rs and the 14-day sampling period.

**Table 1:** Selected reaction monitoring (SRM) conditions used in LC-MS/MS method to determine the seven PFAS of interest and the two isotopically-labeled standards used. MQLs for CPS are also reported. Compounds ordered by fluoro-alkyl chain

PFAS	Formula	Cone voltage (V)	Quantifier transition (m/z)	Collision energy (eV)	Qualifier transition (m/z)	Collision energy (eV)	RT (min)	R^2^	MQLs (ng L^−1^)
PFBS	C_4_F_9_SO_3_^−^	45	299 > 80	30	299 > 99	23	1.38	0.993	1.73
PFHxA	C_6_F_11_O_2_^−^	16	313 > 269	10	313 > 119	24	2.46	0.991	1.46
PFHxS	C_6_F_13_SO_3_^−^	55	399 > 80	31	399 > 99	32	7.02	0.983	1.18
PFOA	C_8_F_15_O_2_^−^	18	413 > 369	11	413 > 169	13	8.82	0.993	1.04
PFOS	C_8_F_17_SO_3_^−^	65	499 > 80	35	499 > 99	35	10.20	0.994	2.27
PFNA	C_9_F_17_O_2_^−^	25	463 > 419	11	463 > 169	22	10.02	0.996	1.16
PFDoA	C_12_F_21_O_2_^−^	20	613 > 569	15	613 > 319	16	11.81	0.994	43.4
^13^C_4_-PFOA	^13^C_4_C_4_F_15_O_2_^−^	20	417 > 372	11	417 > 172	12	8.82	-	-
^13^C_4_-PFOS	^13^C_4_C_4_F_17_SO_3_^−^	65	503 > 80	35	503 > 99	35	10.20	-	-

For groundwater samples, an R script was developed to calculate aqueous concentrations using the experimentally determined Rₛ. Blank contributions, calculated as the average of the blank signals, were subtracted when at least three blank samples showed quantifiable signals. Data points were flagged if outside the calibration range, had signal-to-noise ratios below 10, or exhibited ion ratios deviating by more than a factor of two from those observed in calibration standards.

## Results and discussion

### Calibration of the CPS and comparison with other studies

The accumulation of the seven target PFAS in the CPSs increased linearly with deployment time over the 20-day exposure period. Linear regression of accumulated mass versus time yielded coefficients of determination (R^2^) ranging from 0.79 to 0.83 for six out of seven PFAS compounds indicating an approximately linear accumulation trend (Table 2). Rs varied between 1.51 and 3.30 mL day^−1^. However, PFDoA showed an R^2^ of 0.72 (Table 2) and the Rs was more than one order of magnitude lower, at 0.08 mL day^−1^. Its lower polarity and steric hindrance due to its longer chain are likely the cause of its lesser diffusion through the membrane and/or lower retention in the sorbent. Nonetheless, the 95% confidence intervals of all slopes excluded zero, confirming statistically significant accumulation throughout the calibration period. No evidence of curvature or plateauing was observed (Figure S1), suggesting that samplers operated within the kinetic uptake regime and did not approach equilibrium during the experiment. These results demonstrate that CPS provides reproducible and linear uptake for all investigated compounds under controlled conditions, despite the low sampling rate for PFDoA. However, as the last 3 points of the CPS calibration (16, 18 and 20 days) showed higher variability than initial time points, a period of 14 days was selected for CPS field deployment. The De is also shown in Table 2.

**Table 2:** Calibration parameters for CPSs. Values are shown as estimate with 95% confidence intervals from linear regression of accumulated mass versus exposure time. Parameters are *k* (uptake rate, slope), *R*^*2*^ (coefficient of determination), *Rs* (sampling rate), and *De* (effective diffusion coefficient)

PFAS	k (ng day^−1^) (95% CI)	R^2^	Rs (mL day^−1^) (95% CI)	De (cm^2^ s^−1^) (95% CI)
PFBS	0.99 (0.76–1.22)	0.82	1.98 (1.52–2.43)	2.06 (1.59–2.54) × 10^−7^
PFHxA	1.17 (0.88–1.47)	0.79	2.35 (1.76–2.94)	2.45 (1.83–3.07) × 10^−7^
PFHxS	1.45 (1.10–1.80)	0.81	2.90 (2.20–3.60)	3.03 (2.30–3.75) × 10^−7^
PFOA	1.65 (1.23–2.07)	0.79	3.30 (2.46–4.15)	3.45 (2.57–4.33) × 10^−7^
PFOS	0.75 (0.57–0.93)	0.81	1.51 (1.14–1.87)	1.57 (1.19–1.95) × 10^−7^
PFNA	1.48 (1.14–1.82)	0.83	2.96 (2.29–3.63)	3.09 (2.39–3.79) × 10^−7^
PFDoA	0.04 (0.03–0.05)	0.72	0.08 (0.05–0.10)	8.26 (5.69–10.8) × 10^−9^

Comparison with literature data (Table 3) indicates that Rs for CPSs are one to two orders of magnitude lower than those reported for other passive samplers, with the exception of ceramic filter tubes (CFT), which are also based on ceramic dosimeter principles similar to CPSs but have much lower Rs. These differences are mainly attributed to the dimensions of the different passive samplers. The highest Rs were observed for POCIS, which use a polyethersulfone membrane of 0.1 mm, allowing a high diffusivity of the contaminants. Among POCIS, most of the studies use Oasis HLB sorbent, but the highest Rs were observed with X-AW sorbent, which allows a very effective anion exchange retention. Weak Anion exchange and Oasis HLB performed in a very similar way, but high variations were observed among studies. DGT had also relatively high Rs, attributed also to the membrane wall thickness. Lower sampling rates result in reduced analytical sensitivity but allow for longer deployment times. Moreover, the increased membrane thickness is expected to limit the influence of water flow on the sampling rate, an effect that has been reported to be significant for POCIS samplers [15, 17].

**Table 3:** Sampling rates (*Rs*, mL day^−1^) and standard error (when available) for 7 target PFAS in CPSs in the configuration with 300 mg Oasis HLB and 100 mg Porapak RXN RP and other passive samplers reported in the literature. The line *time* describes the number of days of the calibration experiment and, in brackets, the days of field deployment described in each study

PFAS	CPS (HLB + Porapak)	POCIS^a^ (HLB)	POCIS^b^ (HLB)	POCIS^c^ (HLB)	POCIS^a^ (WAX)	POCIS^d^ (WAX)	POCIS^b^ (ILL)	POCIS^e^ (X-AW)	PE tube^f^ (HLB)	Steel rings^g^ (HLB)	CFT^h^ (WAX)
PFBS	1.98 ± 0.11	28 ± 6	–	–	40 ± 9.1	–	–	370 ± 70	16 ± 5.2	24.8 ± 0.6	0.27
PFHxA	2.35 ± 0.14	29 ± 12	78	73	32 ± 7.2	5.0 ± 0.3	52	290 ± 50	14 ± 3.1	72.3 ± 9.9	0.26
PFHxS	2.90 ± 0.17	46 ± 12	72	–	49 ± 11	–	32	–	15 ± 10	72.0 ± 1.9	–
PFOA	3.30 ± 0.20	61 ± 14	93	117	62 ± 12	6.0 ± 0.4	92	160 ± 10	20 ± 3.2	48.3 ± 1.5	0.29
PFOS	1.51 ± 0.09	88 ± 12	63	147	71 ± 12	–	28	360 ± 80	17 ± 3.6	37.8 ± 1.2	0.13
PFNA	2.96 ± 0.16	77 ± 16	–	34	70 ± 9.7	11 ± 0.6	–	230 ± 30	28 ± 4.0	40.3 ± 1.3	–
PFDoA	0.08 ± 0.01	38 ± 8	69	–	33 ± 5.6	6.0 ± 1.0	8.1	–	–	4.9 ± 0.2	–
Time (days)	20 (14)	28 (14)	21 (7)	20	28 (14)	14 (10)	21 (7)	26 (7)	29 (21)	16 (28)	3 (7)

^a^Gobelius et al. [12],. POCIS with 200 mg of HLB or WAX sorbent. ^b^Wang et al. [13], POCIS with 30 mg of HLB or ILL sorbent. ^c^Fedorova et al. [14], POCIS with 200 mg HLB sorbent. Calibration directly in wastewater effluent. ^d^Li et al. [15], POCIS with 200 mg HLB sorbent, stirring velocity 0.085 m s^−1^. ^e^Kaserzon et al. [18], POCIS with 600 mg Strata X-AW sorbent. ^f^Gardiner et al. [19], Polyethylene microporous tubes (PE) with 600 mg HLB sorbent. ^g^Urík and Vrana [20], Stainless-steel rings with HLB sorbent. ^h^Cheng et al. [21], Ceramic filter tubes (CFT) with WAX sorbent dispersed in a hydrogel rod

Results shown in Table 3 indicate the suitability of the several passive samplers for the retention of PFAS from water under laboratory-controlled conditions. In our study, the concentration in water from the CPS calibration experiment was 500 ng L^−1^, chosen to simulate the low concentration in which PFAS can occur in groundwater. The CPS performance showed a linear uptake with time, resulting in Rs that allowed their deployment over a period up to 20 days without saturation. Nonetheless, the CPSs were deployed for a shorter time in the field (14 days), in order to exploit the window in which accumulation is more linear (Figure S1) and for practical reasons related to sampling, i.e. allowing for leeway in case CPS retrieval had to be delayed due to unforeseen circumstances such as weather conditions. In addition, the combination of Oasis HLB and Porapak provided a high retention efficiency, allowing a high enrichment factor. This configuration was thereafter used for monitoring PFAS in Besòs River groundwater and river water.

### Background assessment and quality control

Potential sources of PFAS background contamination associated with laboratory materials, solvents, calibration procedures, and field handling were systematically evaluated using the blank screening experiments described in “Field Deployment in the Besòs River Basin”.

No target PFAS were detected above detection limits in extracts of CPS caps, glass vials and caps, aluminum foil, nitrile gloves, pipette tips, or solvent bottle septa. Similarly, procedural blanks prepared from methanol taken directly from the original bottle and from laboratory solvent dispensers showed no detectable PFAS signals.

The blank calibration experiment, conducted using CPSs exposed to unspiked bottled water under calibration-relevant conditions, showed no measurable accumulation of five out of seven target compounds over the 20-day exposure period. For PFHxA, a minor time-dependent accumulation was observed, reaching less than 1 ng after 20 days, corresponding to less than 3% of the mass accumulated during the calibration experiments. Given its negligible contribution relative to the analytical signal, this background uptake was considered insignificant and no blank correction was applied. For PFBS, a constant background mass of approximately 0.5 ng was observed both in bulk sorbent extracts and in CPSs retrieved from the blank experiment. As this contribution was independent of deployment time, it represents a constant offset and does not influence the slope of the uptake curve. Consequently, it does not affect the estimation of Rs or De, and was therefore not corrected for in subsequent calculations.

Field blank data were evaluated on a compound-specific basis. For PFHxS, PFNA and PFOA, no blank subtraction was applied as field blank concentrations were below the MQLs. For PFBS, PFHxA and PFOS, background contamination was observed in field blanks with average blank concentrations of 34.4 ng L^−1^, 22.1 ng L^−1^ and 7.65 ng L^−1^, respectively. Therefore, their average value was subtracted from the measured concentrations in field samples.

### PFAS measurement in groundwater through CPSs

**Fig. 2:**
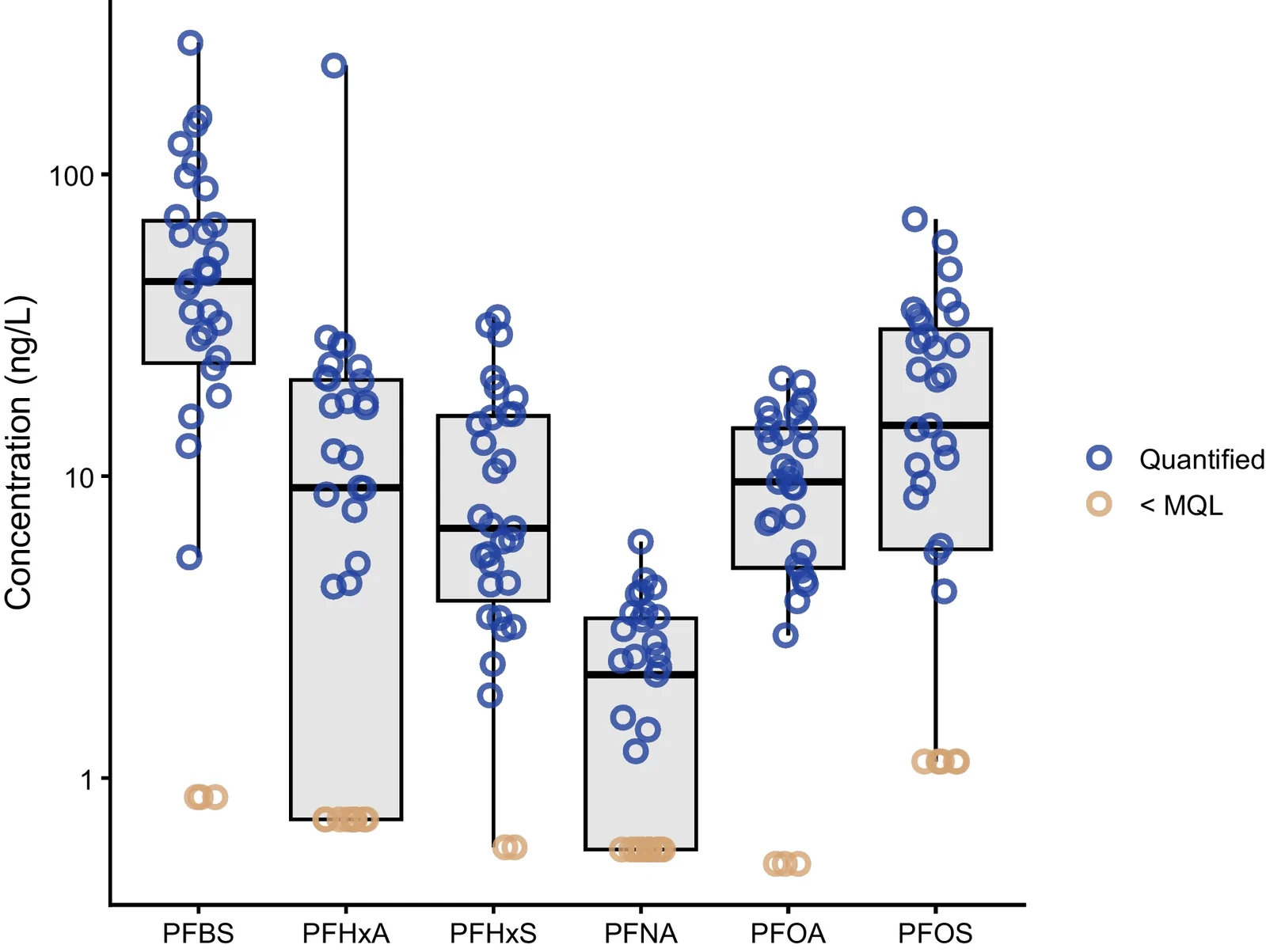
PFAS concentrations across sampling locations shown as box-and-whisker plots. Boxes indicate the interquartile range, and the central line represents the median. Whiskers extend to 1.5 × IQR, and outliers are plotted individually. Light-colored circles denote values below the method quantification limit (MQL)

**Fig. 3:**
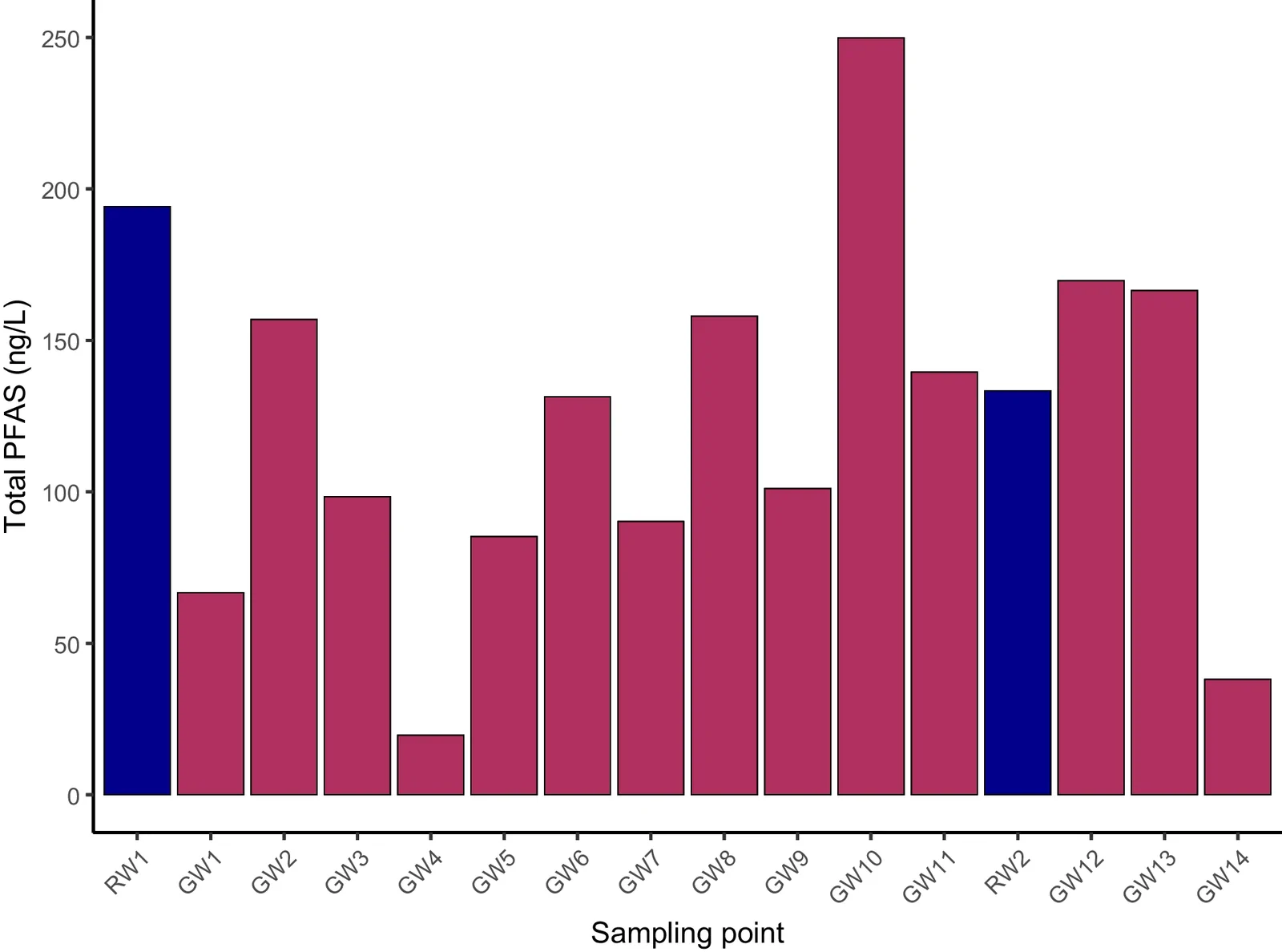
Total PFAS concentrations (ng L^−1^) across sampling locations (from upriver to river mouth, see map in Fig. 1), as the sum of the concentrations of PFBS, PFHxA, PFHxS, PFOA, PFOS and PFNA

PFAS were detected at all sampling locations along the Besòs River (Fig. 2), indicating widespread contamination. Figure 2 shows that there is a relatively low variability in the concentrations detected in CPS duplicates and in all wells, in agreement with the fact that concentrations of contaminants in groundwater do not vary largely. According to median values, the main PFAS detected were PFBS > PFOS > PFOA > PFHxA > PFHxS > PFNA, and PFDoA was never detected. The absence of PFDoA was attributed to its substantially lower sampling rate, which likely resulted in insufficient accumulation during the 14-day deployment period. Also, it is less soluble than other PFAS, and thus, it has lower chances of being present in GW. The lowest detection frequency was for PFHxA and PFNA (Fig. 2). The sum of measured PFAS concentrations ranged from 19.7 ng L^−1^ (GW4) to 250 ng L^−1^ (GW10), with concentrations varying among sites (Fig. 3). No clear relationship was observed between total PFAS concentrations and proximity to the river or upstream–downstream position. Sites with the lowest concentrations were GW4 and GW14, while GW10 had the highest concentration. The concentration in the 2 river waters was very similar. Seven groundwater sampling points and the two river waters exhibited concentrations higher than 100 ng L^−1^, which is the limit set by the EU from 2024 for drinking water [27]. This is relevant because the Besòs aquifer is expected to be exploited to provide drinking water to the Barcelona city.

**Fig. 4:**
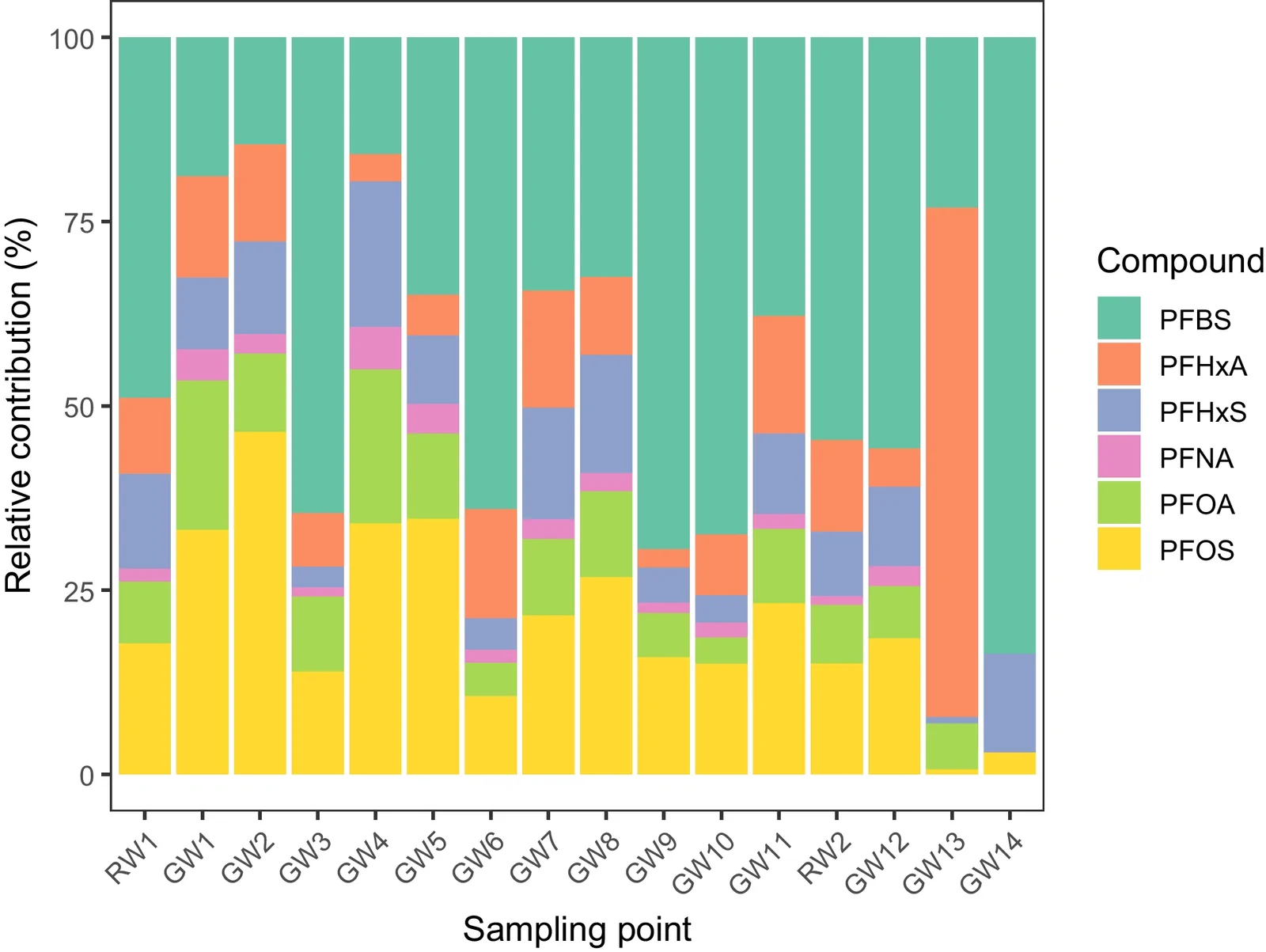
Relative contribution of the target PFAS to the total PFAS measured

The contamination profile was dominated by PFBS and PFOS, whereas PFNA had a low contribution to total PFAS (Fig. 4). The relative contribution of individual PFAS varied among sampling locations, reflecting differences along the aquifer. The 2 river waters analysed (RW1 and RW2) despite being around 12 km distance have a very similar patterns, indicating Besòs river background pollution profile. Samples GW3, GW6, GW9, GW10 and GW12 have a similar profile than river waters, suggesting hydraulic connectivity between river water and GW. For these samples, PFBS accounted for approximately 50% of total PFAS contribution. This pattern may be consistent with the prevalent use and environmental release of short-chain PFAS as alternatives to legacy compounds such as PFOS and PFOA. Samples GW1, GW2, GW4, GW5, GW7, GW8 and GW11 had similar profiles with a larger contribution of PFOS, PFOA and PFHxS compared to the other samples, suggesting maybe interactivity among these wells affected by similar pollution source, although at this stage it is impossible to identify it. GW13 had a high contribution of PFHxA while GW14 had a profile dominated by PFBS. These 2 wells are the closest to the sea and might be affected by different pollution sources.

Several studies have modelled the natural groundwater recharge of the Besòs aquifer, based on dissolved ions [28] or contaminants of emerging concern [29, 30]. Sewage discharge, rainfall, leakage from the urban water system, urban runoff, and infiltration from the Besòs River were identified as the main contributors to groundwater recharge. These studies suggest that contaminants are primarily transferred from surface water to groundwater through river infiltration processes. In the present study, PFAS concentrations in groundwater and river water were of the same order of magnitude, and no systematic concentration gradient between river water and groundwater was evident. Therefore, while surface water–groundwater interactions likely play a role in PFAS occurrence, further studies are needed to identify pollution sources and confirm river-to-aquifer transfer as the dominant contamination pathway.

The concentrations measured in this study are consistent with those reported in a previous investigation of the Besòs River and adjacent groundwater using grab sampling and solid-phase extraction with WAX cartridges. That study reported a mean PFBS concentration of 109 ng L^−1^and the ubiquitous presence of PFBS, PFHxA, PFHxS, PFOA, PFOS and PFNA across all samples [31]. Furthermore, the levels observed in the present study are comparable to those reported for other European groundwaters impacted by anthropogenic activities. For example, a survey in Sweden reported a mean ∑_26_PFAS concentration of 49 ng L^−1^, although the median concentration was substantially lower (0.04 ng L^−1^), reflecting the presence of localized contamination pollution gradients. In that study, a maximum concentration of 6400 ng L^−1^ was observed near a firefighting training site, indicating a pollution hotspot affecting the quality of the aquifer [32]. A large-scale review of more than 100 European rivers reported mean and median PFOS concentrations of 39 ng L^−1^ and 6 ng L^−1^, respectively, with a detection frequency of 94%, and PFOS was frequently reported at higher concentrations than PFOA [33]. However, in the present study we found the predominance of PFBS, which is consistent with its short carbon chain length, which confers higher mobility in aquatic environments and facilitates transport to groundwater. It fits the findings from the previous Besòs study, which found a contamination profile dominated by short-chain PFAS [31].

Overall, PFAS concentrations measured in the Besòs River are comparable to or higher than those reported for other impacted European rivers and substantially exceed typical background levels documented for surface waters. These findings demonstrate the suitability of CPS passive samplers for capturing time-integrated PFAS contamination and highlight the need to monitor the occurrence of PFAS in a systematic way to protect GW resources.

## Conclusions

This study demonstrates the suitability of CPS for the quantitative monitoring of PFAS in GW and surface waters. The sampler was successfully calibrated for seven target compounds and exhibited linear uptake over the 20-day calibration period, confirming its applicability for time-integrated concentration measurements. Laboratory blank experiments enabled the identification and correction of background contamination, ensuring reliable interpretation of field data.

Application of the CPS samplers in the aquifers of the lower course of the Besòs River revealed elevated PFAS concentrations in all wells, with total concentrations ranging from 19 to 250 ng L^−1^, indicating widespread contamination. The concentrations measured in this study are consistent with values previously reported for the Besòs River and comparable aquatic systems using conventional grab sampling approaches. Despite lower sampling rates compared with some existing passive samplers, the CPS provided coherent and environmentally relevant concentration estimates under real field conditions. Overall, these results confirm the CPS sampler as a robust and effective tool for PFAS monitoring and support its use in long-term, integrative assessments of GW contamination.

## Supplementary Information

Below is the link to the electronic supplementary material.

ESM 1 (DOCX 523 KB)

## Data Availability

Data will be made available upon request.
