# Supplementary material for: Performance of ceramic passive samplers for the monitoring of PFAS in surface water and groundwaters
Source: Anal Bioanal Chem. 2026 May 8;418(18):6075–85. doi: 10.1007/s00216-026-06533-y (PMC13541841; doi:10.1007/s00216-026-06533-y)
Supplement: Supplementary file 1 [file 216_2026_6533_MOESM1_ESM.docx]

# Supplementary information

**Performance of Ceramic Passive Samplers for the Monitoring of PFAS in Surface Water and Groundwaters**

Giacomo Moro¹ ², Sergio Santana-Viera^1^, Sandra Pérez¹, Silvia Lacorte¹*

*¹ Department of Environmental Chemistry, Institute of Environmental Assessment and Water Research (IDAEA-CSIC), Barcelona, Spain*

*² Doctoral Programme in Analytical Chemistry and the Environment, University of Barcelona, Barcelona, Spain*

* Corresponding author Silvia Lacorte (silvia.lacorte@idaea.csic.es)

**Figure S1.** Calibration curves of the ceramic passive samplers for the seven target PFAS. Grey points represent individual replicates, while black points indicate mean values. Solid lines correspond to linear regressions.

**Figure S2.** Residual plots of the ceramic passive sampler calibration for the seven target PFAS. Black dots indicate individual replicates.
